# Umbilical Cord and Neonatal Transthyretin and Their Relationship to Growth and Nutrition in Preterm Infants

**DOI:** 10.5041/RMMJ.10470

**Published:** 2022-04-26

**Authors:** Clair Habib, Irit Maor, Irit Shoris, Svetlana Tsuprun, David Bader, Arieh Riskin

**Affiliations:** 1 Genetics Institute and Pediatric Metabolic Unit, Rambam Health Care Campus, Haifa, Israel; 2 Rappaport Faculty of Medicine, Technion, Israel Institute of Technology, Haifa, Israel; 3 Department of Neonatology, Bnai Zion Medical Center, Haifa, Israel; 4 Biochemistry Laboratory, Bnai Zion Medical Center, Haifa, Israel

**Keywords:** Growth, nutritional status, prealbumin, transthyretin (TTR), umbilical cord

## Abstract

**Background:**

Transthyretin (TTR), also known as prealbumin, has been suggested as an indicator of protein and nutritional status.

**Objective:**

The aim of this study was to examine the maternal and umbilical cord (UC) TTR in relation to intrauterine growth, and the serum TTR of preterm infants in relation to nutritional status and growth.

**Methods:**

After application of exclusion criteria, 49 preterm infants (mean gestational age and birth-weight 32.9±2.9 weeks and 1822±556 g) were included in the study. Transthyretin was sampled at birth and on days 14, 28, and at discharge with growth parameters and nutritional laboratory test results.

**Results:**

Mean UC and maternal TTR were positively correlated (8.5±2.4 mg/dL and 20.4±7.0 mg/dL, *r*=0.31, *P*=0.07). Umbilical cord TTR was neither an index of maturity nor of intrauterine growth. Umbilical cord TTR was higher in females (9.4±2.6 versus 7.6±1.8 mg/dL, *P*=0.015). Maternal TTR was lower in twin pregnancies (16.8±4.9 versus 22.5±7.3 mg/dL, *P*=0.007). Although TTR levels gradually increased over time in correlation with post-menstrual and chronological ages (*r*=0.24, *P*=0.011 and *r*=0.40, *P*<0.001, respectively), there was no correlation to weight gain (*r*=0.10, *P*=0.41), nutritional status, protein intake, or nutritional laboratory test results. The only significant correlations were between TTR and glucose and triglycerides levels (*r*=0.51, *P*<0.001 for both).

**Conclusions:**

Although TTR levels increased over time, we could not demonstrate significant correlations between TTR and indices of the nutritional status in preterm infants at birth or during the neonatal course.

## INTRODUCTION

Growth and nutrition are important factors that significantly impact pregnancy and neonatal outcomes, especially in restricted intrauterine growth and premature infants. In recent years, significant effort has been made to provide neonatal care focused on optimal nutrition for growth in premature infants by combining parenteral and enteral nutrition. However, beyond growth parameters, sufficient laboratory nutritional indices are lacking that could help to monitor and optimize neonatal nutritional support. Transthyretin (TTR; also known as prealbumin) is a plasma protein secreted mainly by the liver.^1^ It is involved in the transport of thyroid hormones and indirectly in the carriage of vitamin A through the mediation of retinol-binding protein (RBP). Transthyretin acts as a binding protein for thyroxine and RBP. It has been suggested that TTR may be a biochemical indicator of protein deficiency and used as a marker of nutritional status.^1^,^2^ The TTR serum concentration reflects liver synthesis capacity and is markedly diminished in malnutrition. Its serum half-life is approximately 2 days (as compared to albumin with a serum half-life of up to 20 days). Hence, TTR has been suggested as a marker when monitoring nutritional improvement since its serum levels could reflect rapid changes in visceral protein status.^3^ Previous studies have shown TTR to have significant positive correlation with mean protein intake (but not necessarily total energy intake), as well as with weight, length, and head circumference of ill non-infected premature infants. Furthermore, it has been suggested that TTR changes precede the changes in growth parameters, thereby giving clinicians the opportunity to intervene before growth velocity changes occur.^4^,^5^ However, TTR may not be a sensitive marker for evaluating the adequacy of nutritional support in critically ill patients with infection or inflammation, because during inflammation the liver synthesizes acute-phase proteins such as C-reactive protein at the expense of TTR.^2^,^6^,^7^

One study indicated that serum TTR levels are low during fetal life but increase throughout gestation,^8^ positively correlating with increasing gestational age (GA) and birth-weight (BW).^9^ Birth-weight was also correlated to both fetal umbilical cord (UC) and maternal plasma TTR concentrations.^10^ Others have suggested that small-for-gestational age (SGA) newborns had significantly lower TTR levels compared to appropriate-for-gestational age (AGA) matched controls.^9^,^11^ However, prenatal steroid exposure, lung maturity, and postnatal morbidity have been shown to affect TTR levels, very much like inflammation.^6^,^9^

It was also suggested that UC TTR and RBP, although substantially lower than adult concentrations,^8^ could be good markers for determining the nutritional status of newborns, especially as measures of serum protein mass (SPM), which correlate well to anthropometric measures.^7^,^12^ Although TTR serum concentrations have wide individual variability in infants of the same postnatal age,^11^ in the absence of better markers, experts once considered TTR as a relatively good nutritional marker, especially for protein intake.^4^,^5^ One study showed that serum protein mass levels of TTR and RBP in serial nutritional assessments of very-low-birth-weight infants during the neonatal period were better indicators than serial measurements of serum protein levels.^13^ However, in our clinical practice TTR has not been widely used as an indicator of nutritional status in preterm infants, and when used it did not show the above suggested advantages as a helpful marker of nutritional nor of protein status.

This study therefore revisited the use of TTR levels in preterm infants from birth and throughout the neonatal course with the following objectives: (1) to study the relationship between UC TTR and intrauterine growth by comparing the TTR levels between the AGA and SGA groups in relation to prenatal and delivery anthropometric measures; (2) to study the correlation between maternal and UC TTR levels; and (3) to study how well TTR reflects the newborn nutritional status in relation to caloric and other nutrients intakes, anthropometric measures, nutritional laboratory indices, and type of nutrition (human milk, fortification, or formula).

## METHODS

This was a non-interventional clinical prospective observational study from the neonatal intensive care unit (NICU) of Bnai Zion Medical Center, Haifa, Israel. Pregnant women with imminent preterm delivery and whose infants were expected to be admitted to the NICU (i.e. preterm infants at GA <36 weeks and BW <2000 g) were asked to participate in this study. All participants received a full explanation of the study and then signed a consent form. Forty-nine preterm infants (GA <36 weeks, BW <2000 g) were admitted to the NICU over a 3-month period. Infants were excluded from the study if parents declined to participate in the study, or if the infants were born with congenital anomalies that mandated surgical intervention, and/or involved feeding problems and the need for prolonged parenteral nutrition (e.g. gastrointestinal atresia or obstruction).

This study was approved by the ethics committee of the Bnai Zion Medical Center (#014515 BNZ).

### Data Collection

Data were collected regarding the infants’ prenatal history, at delivery, and in the NICU (days 14, 28, and discharge home). Prenatal history data included pregnancy type: spontaneous or induced (fertility treatments), prenatal tests and imaging (ultrasound), intra-uterine growth parameters, and, if intrauterine growth restriction (IUGR) was suspected, the screening and evaluations performed to determine the possible cause. At delivery, the data included the type of delivery (spontaneous, assisted, or cesarean), singleton or multiple births, GA, sex, BW, AGA or SGA, and head circumference (fronto-occipital circumference). Data collected in the NICU related to clinical course, morbidities, treatments, growth parameters (weight, fronto-occipital circumference), nutrition (parenteral, enteral), mother’s milk, enrichment (human milk fortifier or enriched preterm formula), and nutritional and caloric assessment (based on the infant intake, including sampling of mother’s milk for protein, calcium, and phosphorus).

### Laboratory Studies

At delivery, TTR (prealbumin) was concomitantly sampled from the UC and maternal blood. The TTR was sampled again in the NICU at 14 and 28 days and at discharge home. The TTR assay is an immunoturbidimetric procedure that measures increasing sample turbidity caused by the formation of precipitate when specific antibody to TTR is added to human TTR.^7^ Commercial immunoturbidimetric assay kits are available for measuring TTR serum levels. The Reagent Kit PREA (Cobas Integra PREA, Cat no. 20764655322; Roche Diagnostics, Basel, Switzerland) used in this study was supplied as a liquid, ready-to-use-for-analysis using the Roche COBAS 6000 analyzer (Roche Diagnostics, Basel, Switzerland).

Routine serum indices of metabolic and nutritional status were measured at ages 14 and 28 days and at discharge, as part of the clinical follow-up. These included glucose, mineral status markers (i.e. calcium, phosphorus, magnesium, and alkaline phosphatase), electrolytes (sodium, potassium, and chloride), and the acid–base status in the blood gasses (pH and base excess [bicarbonate]). Urea, total protein, albumin, and TTR were measured as markers of protein status. Triglyceride and cholesterol levels were measured to determine lipid status. Liver function tests were performed by total and direct bilirubin and liver enzymes (aspartate aminotransferase, alanine transaminase, and gamma glutamyl transpeptidase). Renal function tests included urea and creatinine. The complete blood count and blood precursor’s metabolism, including iron, transferrin, ferritin, folic acid, and vitamin B12 serum levels, were also checked.

### Data Analysis

Data were recorded in an Excel spreadsheet (Microsoft Office, Seattle, WA, USA) and statistically analyzed using SigmaPlot, version 11.0 (Systat Software Inc., San Jose, CA, USA) and Minitab®, version 16.2.2 (Minitab Inc., State College, PA, USA, and Coventry, UK). Statistical analysis included descriptive statistics, Student’s *t* test, or Mann–Whitney rank sum test for comparison of continuous variables according to their distributions, chi-square test for comparisons of categorical variables, linear regressions, and Pearson’s correlation coefficient. *P* values of less than 0.05 were considered statistically significant.

## RESULTS

Data were collected from 49 preterm infants that met the inclusion criteria: 26 (53%) males and 23 (47%) females (mean GA, 32.9±2.9 weeks [24.4–37.9 weeks]; mean BW, 1822±556 g [810–2780 g]). There were no significant differences in GA and BW between males and females (32.7±2.9 versus 32.7± 3.3 weeks, and 1845±587 versus 1777±534 g, respectively).

### Umbilical Cord and Maternal TTR

The mean UC TTR was 8.5±2.4 mg/dL (3.6–14.5 mg/dL), and the mean maternal TTR was 20.4±7.0 mg/dL (8.5–39.4 mg/dL). The UC TTR and maternal TTR were not correlated to GA or BW. Infant UC TTR levels were significantly higher in females (9.4±2.6 versus 7.6±1.8 mg/dL, *P*=0.018), although there were no significant differences in GA or BW between sexes. However, there were no similar differences in mean maternal TTR for offspring by sex (males, 20.73±7.1; females, 19.9±7.2; *P*=0.738).

The UC TTR levels were similar in SGA infants and non-SGA infants (8.38±1.99 versus 8.74±2.5 mg/dL, respectively; *P*=0.909); this was also true for maternal TTR (19.08±8.8 versus 20.5±6.8 mg/dL, respectively; *P*=0.710). Some of the SGA infants were defined as IUGR during pregnancy, and their UC TTR levels were slightly higher compared to preterm infants without a history of IUGR (9.2±2.4 versus 8.3±2.4 mg/dL, respectively), but this difference was not statistically significant (*P*=0.34). This was also true for maternal TTR levels (21.3±6.6 in IUGR versus 20.1±7.2 mg/dL, respectively; *P*=0.68).

**Figure 1:**
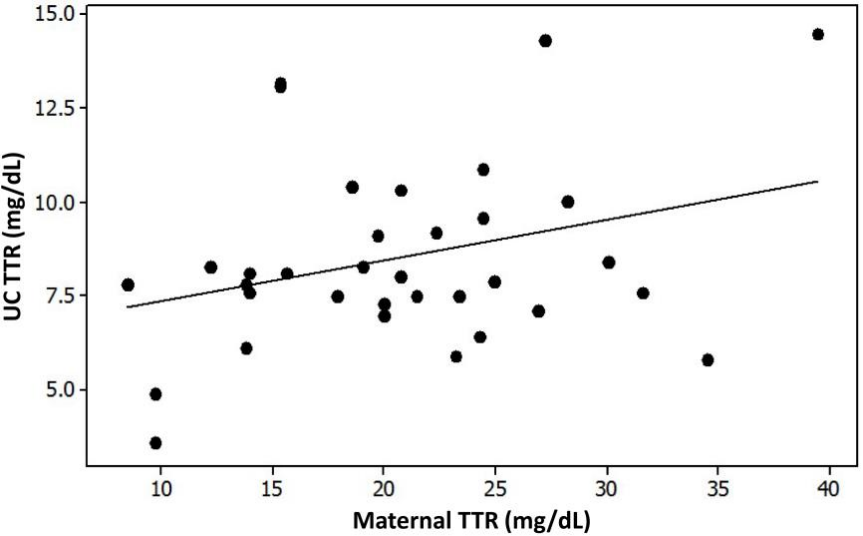
The Correlation between Umbilical Cord (UC) and Maternal Transthyretin (TTR) Serum Levels. Transthyretin levels are in mg/dL. Pearson *r*=0.31, *P*=0.07.

Although there was some correlation between UC and maternal TTR (Pearson *r*=0.31, *P*=0.071), it was not statistically significant and with borderline Pearson *r* (Figure 1). Both maternal and UC TTR levels were unaffected by gestational morbidities, prenatal steroids, premature or prolonged rupture of membranes, or mode of delivery, as shown in Table 1. However, maternal (but not UC) TTR levels were significantly lower in twin pregnancies compared to singleton pregnancies (16.8±4.9 versus 22.5±7.3 mg/dL, respectively; *P*=0.009).

**Table 1:** Umbilical Cord and Maternal Transthyretin and Maternal Correlations with Prenatal Morbidities.

Morbidity	Umbilical Cord TTR mg/dL			Maternal TTR mg/dL		*P* Value
		
	Mean	SD	*P* Value	Mean	SD	
PROM >18 h	9.24	2.56	0.083	22.25	7.14	0.294
PROM <18 h	7.93	2.19		19.48	7.04	

PET	10.43	3.18	0.277	17.43	6.48	0.418
No PET	8.26	2.28		20.69	7.17	

Prenatal steroids	8.48	2.64	0.236	20.56	7.18	0.719
No prenatal steroids	7.79	1.10		19.73	7.03	

Normal delivery	9.12	2.81	0.212	23.21	9.29	0.192
Cesarean section	8.09	2.11		19.06	5.64	

PET, preeclampsia, toxemia; PROM, prolonged rupture of membranes; SD, standard deviation; TTR, transthyretin.

Maternal TTR serum levels at birth were not correlated to mothers’ breast milk protein content; however, breast milk protein levels were collected after delivery, while the infants were being cared for in the NICU.

### TTR Levels in Preterm Infants over Time

**Figure 2:**
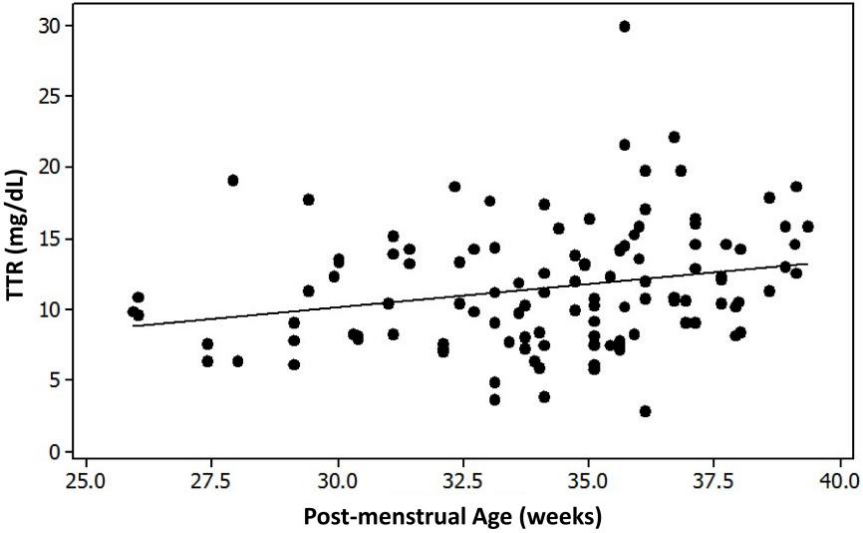
The Correlation between Transthyretin (TTR) Serum Levels and Post-menstrual Age (PMA). Transthyretin levels are in mg/dL. PMA is in weeks. Pearson *r*=0.24, *P*=0.01.

Although there were no significant correlations between UC or maternal TTR and BW or GA, when infant TTR levels were followed over time, they gradually increased. This study found correlations between TTR levels and post-menstrual age (PMA) and chronological age (Pearson *r*=0.24, borderline although significant *P*=0.011, and *r*=0.40, *P*<0.001, respectively) (Figure 2), but TTR was not correlated with weight gain (*r*=0.10, *P*=0.41), albumin (*r*=0.166, *P*=0.294), or caloric or protein intake.

As expected, there was a positive correlation between TTR and transferrin levels (*r*=0.378, *P*=0.015). The TTR levels were negatively correlated with ferritin and folic acid levels (*r*=−0.326, *P*=0.037, and *r*=−0.368, *P*=0.019, respectively).

**Figure 3:**
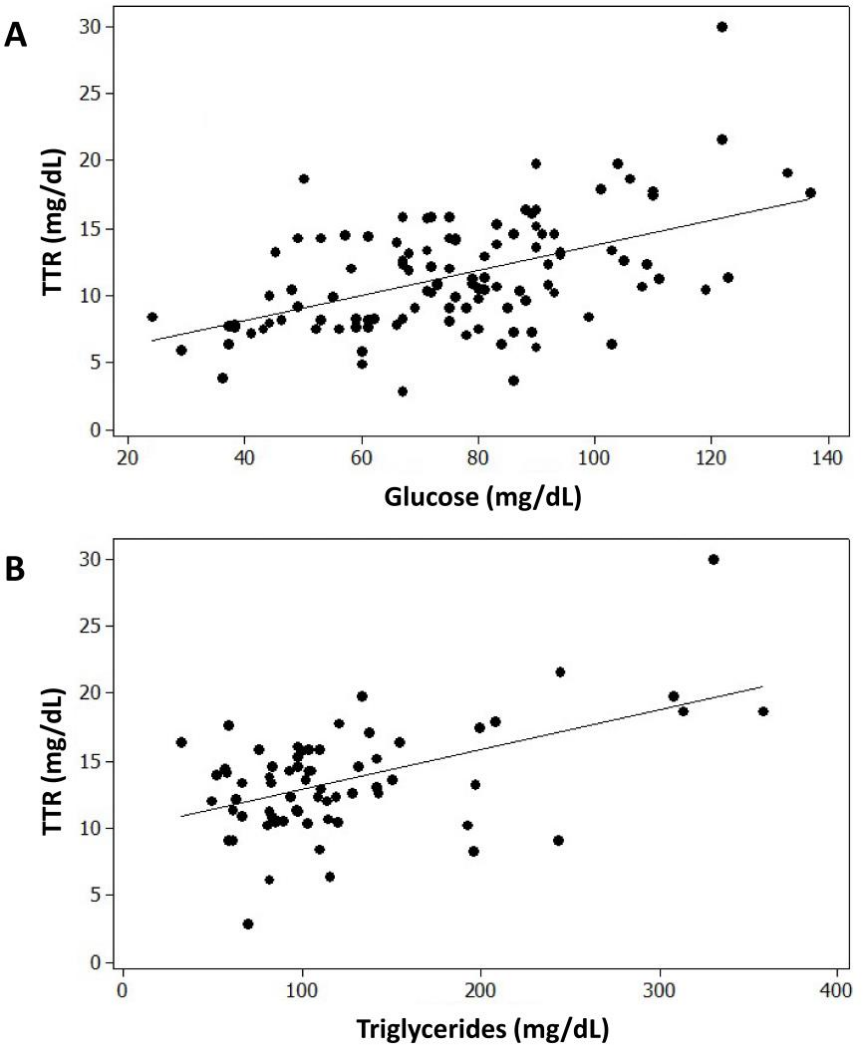
The Correlation between Serum Transthyretin and Glucose (A) and Triglyceride (B) Levels. Transthyretin, glucose, and triglyceride levels are in mg/dL. (Pearson *r*=0.51, *P*<0.001 for both glucose and triglycerides).

Over time, there were no differences in TTR between females and males (11.8±4.2 versus 11.4±4.5 mg/dL, *P*=0.64). Mean TTR levels were still slightly (but not significantly) higher in IUGR infants (13.1± 5.6 versus 11.2±4.0 mg/dL, *P*=0.15). The changes in TTR levels over the NICU course neither correlated with weight gain, nor did they correlate with most nutritional intakes or laboratory parameters. The only significant correlations found were between TTR levels and glucose and triglycerides serum levels (*r*=0.51, *P*<0.001 for both) (Figure 3).

Although correlations between protein intake and urea, total protein, and albumin serum levels (*r*=0.313, *P*=0.008; *r*=0.340, *P*=0.004; *r*=0.303, *P*=0.010, respectively) could have been presented, we could not demonstrate any of the previously suggested TTR correlations neither to these laboratory indices of protein status nor to the calculated protein intake (urea, *r*=−0.055, *P*=0.656; total protein, *r*=0.196, *P*=0.109; albumin, *r*=0.195, *P*=0.110; calculated protein intake, *r*=−0.031, *P*=0.801).

Although the number of days premature infants were on parenteral nutrition correlated with GA (i.e. the younger they were, the longer they needed parenteral nutrition), the TTR levels over time were not correlated to the time preterm infants were on parenteral nutrition (Pearson *r* =−0.017, *P*=0.858).

Although there was a significant correlation between mother’s milk protein content and infant serum urea levels (Pearson *r*=0.682, *P*=0.015), there was no correlation to TTR (*r*=0.027, *P*=0.918).

Infants who were fed mother’s milk (even if fortified) gained less weight compared to infants on formula (mean weight gain with mother’s milk 73.6± 116.4 g, fortified mother’s milk 159.5±111.9 g, formula 171.4±87.4 g, *P*=0.45), but their TTR levels were not significantly different (mother’s milk, 13.73±5.22 mg/dL; fortified mother’s milk, 13.06± 3.2 mg/dL; formula, 14.04±1.62 mg/dL; *P*=0.871).

Weight gain was neither correlated with serum albumin levels or protein levels in mother’s milk (Pearson *r*=−0.184, *P*=0.314), nor with TTR levels at birth or over time (*r*=0.148, *P*=0.116). There was no correlation between TTR levels and weight gain in either AGA or SGA/IUGR infants, but, in any case, good correlations of weight gain to the calculated caloric intake or protein intake in our study group could not be demonstrated.

There was also no correlation of the TTR levels to the complete blood count indices or anemia profile, nor to any biochemical nutritional laboratory markers including acid–base status, liver function tests, and creatinine or mineral status except for chloride and sodium levels (Pearson *r*=−0.346, *P*=0.004; Pearson *r*=−0.238, *P*=0.051, respectively).

We were unable to find any associations between TTR levels and neonatal clinical course (data not shown).

## DISCUSSION

Although TTR levels increased over time, we could not demonstrate significant correlations between TTR and nutritional status indices in preterm infants at birth or during the neonatal course. The results of our study differ from some of the previous studies which suggested that TTR could be considered as one of the biochemical markers of nutritional status.^1^ Previous studies have shown TTR to have a significant positive correlation with mean protein intake (but not necessarily total energy intake), and also with weight, length, and head circumference of ill non-infected premature infants; it was even suggested that the changes in TTR preceded the changes in growth parameters, giving the clinician the opportunity to intervene before changes in growth velocity occur.^3^,^4^

In a recent study, Kim et al.^14^ studied 80 preterm and term infants, and concluded that TTR (referred to by Kim et al. as prealbumin) can be considered as an indicator of sufficient growth in early preterm infants, based on TTR measurements on postnatal day 14 in early preterm infants; they showed significant correlations with anthropometric measurements and calorie intake, but not with protein intake. Transthyretin levels also increased with time in the late preterm and term groups but did not show the same correlations with anthropometric or nutritional intakes. At birth, TTR levels were the lowest in late preterm babies. At postnatal day 28, TTR levels of many preterm infants did not reach those seen in term babies at birth.^14^

Our study found no correlation between TTR levels in preterm babies, at birth or over the NICU course, and growth, i.e. weight gain. We were unable to demonstrate any of the suggested associations between TTR levels and nutritional status, protein intake, or laboratory indices of protein status. These findings address the debate in the medical literature as to whether or not serum protein markers such as TTR are really good indicative markers of nutritional status.^2^,^15^–^17^ Albumin, TTR, transferrin, and RBP are all synthesized in the liver and integrate protein synthesis and degradation over longer periods of time. Traditionally they have been used as part of the nutritional assessment. However, many times these levels are not indicative of the patient’s protein status, and thus are inappropriate for use as an indicator of nutritional status. Some researchers in recent years think that these markers might not be specific or sensitive indicators of nutritional status because they are greatly influenced by the inflammatory responses among other factors. Transthyretin levels may decrease during physiological stress, infection, liver dysfunction, and over-hydration, or they can increase in the setting of renal dysfunction, corticosteroid therapy, or dehydration.^2^ Some recent studies in adult and pediatric populations have demonstrated a poor relationship between such serum protein levels and nutritional status. The levels of serum protein levels such as TTR are often maintained in a chronic malnourished state (such as anorexia nervosa), and are decreased in well-nourished individuals who have experienced a recent stress or trauma.^2^,^15^–^17^ The question has been raised whether, instead of reflecting overall nutritional status, low serum TTR should be regarded as a bio-marker of increased risk of malnutrition, requiring further nutritional assessment. It was suggested that it could be thus used more as a prognostic marker for monitoring patients receiving nutritional support.^17^ Several studies reported a role for TTR in predicting prognosis that could be related to nutritional status and malnutrition in various clinical conditions.^16^ An algorithm that uses TTR has recently been proposed as a practical guide to help clinicians to stratify their sick intensive care patients by risk of complications and outcome. This algorithm states that TTR screening should only be performed when an acute inflammatory state (based on C-reactive protein levels) was excluded. The algorithm was developed for the adult population and defined a threshold TTR level that was associated with increased mortality and morbidity and longer hospitalization. They also suggested a minimal rate for TTR serum levels increase, below which failure of nutritional therapy was defined.^2^,^16^ Based on our findings we believe that such studies in neonates, especially premature infants, are needed. Such studies will abandon the efforts to define the direct relationship between TTR serum levels and protein or energy intake, nutritional status, and growth parameters, and concentrate instead on trying to re-define TTR serum levels as markers of risk for development of malnutrition and/or poor prognosis.

Although it has been suggested that UC TTR might be an indicator for intrauterine nutritional status, we found that it was neither an efficient index of maturity nor of intrauterine growth of preterm infants.

Our data showed that infant UC TTR levels were significantly higher in females. Quintela et al.^18^ have demonstrated that estradiol increases TTR mRNA and protein levels in the mouse brain choroid plexus *in vitro* and *in vivo*, as well as increasing TTR expression in the liver and its concentrations in peripheral circulation. This could explain the higher TTR levels in females. Goncalves et al.^19^ showed that although dihydrotestosterone could also induce TTR expression in males, in females TTR expression was increased by both dihydrotestosterone and estradiol. In both sexes, dihydrotestosterone treatment was more effective in raising the TTR mRNA levels. This could explain why, on follow-up after delivery, TTR levels that were higher at birth in females became similar to males over time, since the TTR levels increased in males.

Umbilical cord TTR was slightly higher in preterm infants with a history of IUGR, and the mean TTR levels were slightly (but not significantly) higher in IUGR infants. Transthyretin is a placental high-affinity thyroid-binding globulin that is involved in maternal thyroid hormone uptake by the fetus. From that perspective, alteration in thyroid hormone profile and insulin sensitivity may be the epiphenomena of growth retardation.^20^ Landers et al. have shown that T4 increases TTR uptake.^21^ These findings could have helped us explain our findings regarding TTR in IUGR/SGA. However, thyroid function tests were not collected from the infants included in this study, thus we can only speculate on this possible explanation.

Maternal TTR levels were significantly lower in twin pregnancies. This might be due to the fact that in a twin pregnancy the mother needs to provide sufficient nutrition to both twins at the same time, which might cause the mother to experience a form of mild malnutrition. Ingenbleek et al.^22^ suggested that a restrictive diet could cause reduction in hepatic TTR mRNA and hence in TTR levels in the circulation.

Landers et al. described TTR expression localized to placental trophoblasts that started as early as 6 weeks of gestation, while from 13–17 weeks of gestation TTR levels were similar to term levels. Transthyretin is also secreted from the trophoblasts into the maternal and fetal circulation, although much more is secreted to the maternal circulation.^21^ Landers et al. also showed that T4 binding stabilizes the TTR tetramer and increases TTR uptake. They suggested that after placental TTR is secreted to the maternal blood stream, it binds to maternal T4 for uptake by the trophoblasts and is then secreted into the fetal circulation,^21^ hence explaining the correlation between maternal TTR and UC TTR. On the other hand, Landers et al. referred to Fruscalzo et al.^23^ who described a dysregulated placental TTR in intrauterine growth restriction and severe early onset of preeclampsia. This might suggest an explanation for the low levels in twins since with multiple fetuses there might be some restriction of fetal growth.

We were unable to demonstrate any association between maternal TTR serum levels and any pregnancy-associated morbidity, such as preeclampsia,^23^ but this might be related to our relatively small sample size, which was under-powered to show this.

The only significant correlations we found were between TTR levels and glucose and triglycerides serum levels. Landers et al.^24^ have suggested that TTR uptake in the hepatocytes is mediated by the low-density lipoprotein (LDL) receptor family. Sousa et al.^25^ have shown that lipoprotein vesicle integrity is crucial for displacement of TTR binding to cellular surfaces. While TTR uptake does not represent TTR-lipoprotein complex internalization, they showed that a receptor-associated protein, which is a multifunctional ligand that binds to members of the LDL receptor family and antagonizes ligand binding activity, inhibits TTR uptake. These associations could possibly explain the described correlation between TTR levels and triglycerides as it was shown that there is a correlation between TTR levels and lipoproteins, and it is known that there is also an association between triglycerides and LDL levels. In addition, Su et al.^26^ have shown that TTR is expressed in pancreatic cells and plays an important role in glucose homeostasis as it regulates glucagon expression. They measured the glucagon levels in TTR knockout mice after insulin injection and after fasting and showed that there was a very small increase in plasma glucagon levels in both conditions. They also showed that the expression of TTR mRNA in pancreatic cells of TTR knockout mice was significantly lower than in wild-type mice. This could explain the correlation between the TTR levels and glucose levels in our infants.

Our study was limited mainly by its sample size, since we were asked by our clinical monitoring committee to discontinue the study after this pilot sample failed to show any of the expected associations in our study objectives. Although our study had a relatively small sample size, this was also the case in some other studies that examined similar questions.^4^,^5^ In addition, generalization of our results could be limited since this was a single-center study, but this was true for others as well.^14^ Another issue is the degree to which our results could have been affected by neonatal morbidities, especially those related to prematurity. Our study was limited by the relatively small number of participants and was not able to adjust for different neonatal courses. Further larger studies with thorough investigation of morbidities are needed to confirm these results.

In summary, although our study has shown some interesting findings regarding TTR in UC and in preterm infants, it failed to demonstrate any significant association of TTR to either intrauterine or extra uterine growth. In addition, our data did not demonstrate any TTR correlations to protein or nutritional metabolic indices.

## Abbreviations

AGA: appropriate for gestational age
BW: birth-weight
GA: gestational age
IUGR: intrauterine growth restriction
NICU: neonatal intensive care unit
RBP: retinol-binding protein
SGA: small for gestational age
TTR: transthyretin
UC: umbilical cord.
